# Bactericidal activity of 3D-printed hydrogel dressing loaded with gallium maltolate

**DOI:** 10.1063/1.5088801

**Published:** 2019-04-29

**Authors:** Stacy Cereceres, Ziyang Lan, Laura Bryan, Michael Whitely, Thomas Wilems, Hunter Greer, Ellen Ruth Alexander, Robert J. Taylor, Lawrence Bernstein, Noah Cohen, Canaan Whitfield-Cargile, Elizabeth Cosgriff-Hernandez

**Affiliations:** 1 Department of Biomedical Engineering, Texas A&M University, College Station, Texas 77843-3120, USA; 2 Department of Biomedical Engineering, The University of Texas at Austin, Austin, Texas 78712, USA; 3 Department of Veterinary Pathobiology, College of Veterinary Medicine and Biomedical Sciences, Texas A&M University, College Station, Texas 77843, USA; 4 Department of Large Animal Clinical Sciences, College of Veterinary Medicine and Biomedical Sciences, Texas A&M University, College Station, Texas 77843, USA; 5 Department of Veterinary Integrative Biosciences, College of Veterinary Medicine and Biomedical Sciences, Texas A&M University, College Station, Texas 77843, USA; 6 Gallixa LLC, Menlo Park, California 94025, USA

## Abstract

Chronic wounds are projected to reach epidemic proportions worldwide because of the aging population and the increasing incidence of diabetes. Despite extensive research, infection remains one of the leading sources of complications in chronic wounds, resulting in improper healing, biofilm formation, and lower extremity amputation. To address the limitations of standard treatments, we have developed a hydrogel wound dressing with self-tuning moisture control that incorporates a novel antimicrobial agent to eliminate and prevent infection. 3D-printing of a hydrogel dressing with dual porosity resulted in a new dressing with greater flexibility, increased water uptake, and more rapid swelling than bulk hydrogel dressings. Additionally, gallium maltolate (GaM) was incorporated into the dressing to investigate the efficacy of this antimicrobial agent. Loading profiles, release kinetics, and the bactericidal activity against *Staphylococcus aureus* (including methicillin-resistant *Staphylococcus aureus*) of GaM were investigated *in vitro* to identify target profiles that supported infection control. Finally, GaM-loaded hydrogel dressings were evaluated *in vivo*, utilizing a murine splinted-wound model that was inoculated with *S. aureus*. In comparison to an untreated control, GaM dressings markedly reduced the wound bacterial load without compromising wound closure rates. Overall, this work demonstrates the utility of a 3D-printed hydrogel dressing as an antimicrobial dressing to control infection in chronic wounds.

## I. INTRODUCTION

Chronic wounds affect approximately 2.4 to 4.5 million people in the United States, and over half of the nontraumatic lower extremity amputations are due to diabetic foot ulcers.^1,2^ The World Health Organization estimates that over 350 million people globally are affected by Type I and II diabetes with 15% of those patients suffering from diabetic foot ulcers.^3,4^ Diabetic foot ulcerations are highly prevalent due to underlying health complications and delayed healing times. The natural wound healing response to injury occurs in 4 different phases: hemostasis, inflammation, proliferation, and remodeling. In chronic wounds, issues with infection, excessive inflammatory responses, biofilm development, and the inability of cells to respond appropriately to reparative chemotactic factors prevent the phases of wound healing from occurring.^5^ Despite several advances in wound healing to improve healing capability, reduce amputations, and improve patient comfort and care, there is still a need for a multifaceted dressing that can address the complex wound environment.

Ideally, wound dressings would initiate and manage wound healing following the wound healing cascade: manage infection, establish wound fluid balance, and encourage cellular migration to promote healthy tissue formation. Clinical wound dressings can be separated into two different approaches: passive and active wound healing. Passive wound healing options consist of cotton wool, compression bandages, and natural or synthetic gauzes.^6^ Although these traditional passive wound healing options are cost effective, they lack the ability to provide cellular cues to initiate the wound healing process. Many of these dressings also dehydrate the wound bed and can cause further tissue damage during dressing changes.^7^ Active wound healing options such as Integra™, Dermagraft^®^, and OrCel^®^ contain bioactive factors or human-derived cells to provide bioactive factors to target cellular interactions.^8,9^ These commercially available skin substitutes have their own unique benefits, but common problems exhibited are reduced vascularization, decreased biocompatibility, low closure rates, and increased product costs.^8–12^ In addition to their high costs, these dressings also have the potential for graft rejection due to allogenic cells. Despite the advancement of these wound dressings, colonization due to infection is among the most common complications with chronic wounds, increasing healing times and causing damage to the surrounding healthy tissue.^5,13–15^

Although extensive research on infection control has been done, several debated issues still exist: critical wound colonization and the role of biofilm, antimicrobials, and antibiotics.^5^ Attempts at infection control and reduction of inflammatory byproducts have been investigated through debridement. This process helps to remove the necrotic or infected tissue that slows down the wound healing process.^6^ Other protective dressings thought to address infection include absorbent dressings, autolytic debridement dressings, and antimicrobial dressings.^6,16,17^ There has been a large shift in antimicrobial investigation due to the development of antibiotic-resistant bacteria.^18,19^ Zubair *et al.* investigated isolated bacteria from diabetic foot ulcer patients and found several classes of antibiotics susceptible to resistance.^20^ The ability of antimicrobials to be loaded into dressings and delivered topically helps to reduce negative systemic effects. However, clinically available antimicrobial dressings such as iodine and silver have potential for severe negative outcomes. Iodine products are commonly used in wound care to reduce bacterial growth as they have been shown to prove effective against most micro-organisms and disrupt mature biofilms *in vitro*.^21^ Dressings containing iodine, however, are contraindicated for patients suffering from thyroid disorders, Grave's disease, and patients who are pregnant or lactating due to systemic absorption.^22,23^ Silver has been highly investigated as an antimicrobial because it has been shown to be effective against a broad range of micro-organisms.^24,25^ Silver is absorbed by sensitive strains of bacteria impairing cell walls, inhibiting respiration, and inactivating bacterial DNA and RNA.^26^ However, it has been suggested that uncontrolled use of silver could result in bacteria developing resistance and reported incidents of allergic response has occurred.^25,27^ One of the main challenges is the ability to maintain high enough concentrations of silver to provide bactericidal effects without the development of dose- or concentration-dependent toxicity.^10^ In addition to synthetic compounds, antimicrobial peptides have been studied for over two decades and have displayed efficiency in controlling bacterial infection and disrupting biofilm formation.^28^ Antimicrobial peptides can be extracted from natural sources of both prokaryotes (e.g., bacteria) and eukaryotes, such as tyrocidines produced by *B. brevis*, aurelin from jellyfish, fish hepcidins, and human defensins found in neutrophils.^29–32^ Researchers have also synthesized antimicrobial peptide mimics such as pexiganan that can efficiently control the infection with a reduced manufacturing cost.^33,34^ However, the wide application of antimicrobial peptides and synthetic mimics is still limited due to susceptibility to proteases, potential cytotoxicity to human cells, lack of selectivity against some strains, and development of bacterial resistance.^35^

Recently, gallium maltolate (GaM) has been shown to prevent bacterial growth and colonization.^36–39^ GaM is a coordination complex of gallium and maltol, which has an octonal: the water partition coefficient of 0.41, illustrating its solubility in both water and lipids ideal for bacterial uptake.^40,41^ GaM has been found to significantly reduce the number of colony forming units (CFUs) of several different bacteria types often known to cause biofilm formation.^37–39,42^ Gallium functions as a ferric iron mimic that has been used to inhibit various microorganisms by taking advantage of the iron-dependence in bacterial growth.^41^ The impact of GaM on cellular actions is unclear, but gallium has been shown to promote collagen synthesis, cell migration, and favorably modulate integrin expression, which are all important aspects of wound healing.^43,44^ As a result, GaM has the potential to serve as an improved antimicrobial with reduced adverse effects and enhanced wound healing.

The aim of this study was to evaluate the efficacy of a 3D-printed hydrogel dressing loaded with GaM to prevent bacterial infection of chronic wounds. Minimum inhibitory and bactericidal concentrations of GaM were determined to validate its use as an antimicrobial agent. Poly(ethylene glycol)-diacrylate (PEGDA) hydrogel dressings were then fabricated by 3D-printing hydrocolloid inks into a hydrogel dressing with the hierarchical porosity.^45^ PEGDA was selected as the initial hydrogel chemistry due to its well-established biocompatibility, high water absorption, tunable mechanical properties, and photopolymerization which is amenable to our cure-on-dispense printing.^46,47^ These features make PEGDA a better candidate for these wound dressings than poly (vinyl alcohol) (PVA), which is frequently used in wound dressings and wound management systems.^48^ Although PEGDA was selected for this initial study, we have previously demonstrated the versatility in generating hydrocolloid inks from a variety of hydrogel chemistries.^45^ We hypothesized that the ability to control hydrogel properties, emulsion variables, and dressing geometry will allow for the development of a tunable dressing with a potential to improve wound moisture balance. The PEGDA hydrogel dressings were expected to display high water uptake and rapid, self-tuning hydration due to the dual porosity structure achieved with this approach. To test this hypothesis, the effect of this templated architecture on hydrogel water uptake and swelling rate was characterized in comparison to bulk hydrogels. It has been shown that appropriate fluid balance improves wound healing by preventing tissue dehydration and cell death, accelerating angiogenesis, increasing the breakdown of dead tissue, and enhancing the interaction of growth factors with target cells.^7^ GaM-loaded hydrogels were then characterized using UV-Vis to determine release profiles at two loading levels. Finally, *in vivo* GaM release, bactericidal effects, wound closure, and host response were evaluated to determine its potential as an antimicrobial wound dressing. Overall, the goal of this work was to investigate GaM as an antimicrobial agent for wound care and demonstrate its therapeutic application in a topical wound dressing.

## II. RESULTS

### A. Characterization of 3D-printed hydrogel dressing

**FIG. 1.**
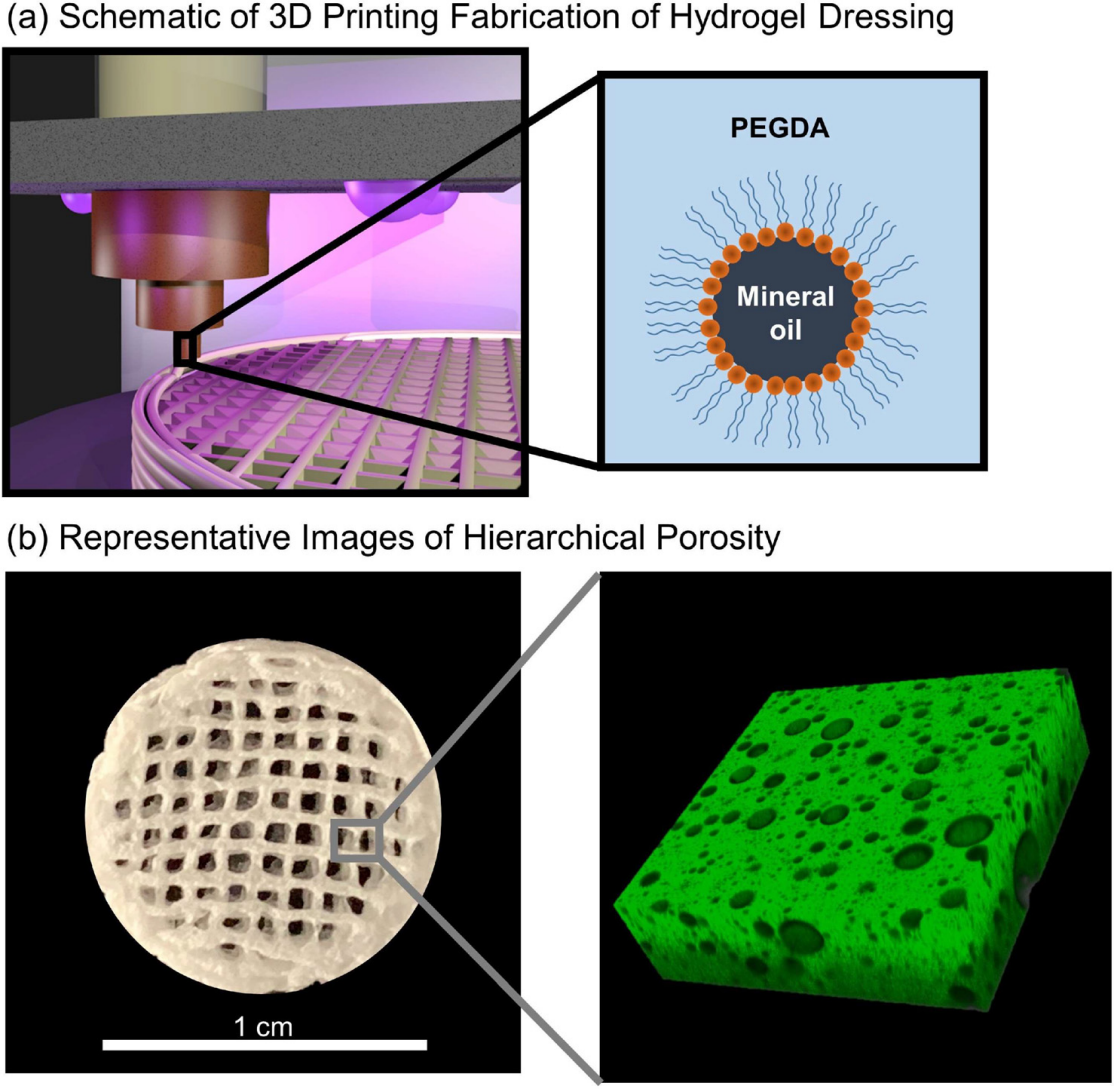
(a) Schematic representation of 3D-printed hydrogel dressing fabrication from extrusion deposition of hydrocolloid ink (mineral oil droplets dispersed in aqueous solution of PEGDA) with UV cure-on-dispense. (b) A representative image of 3D-printed hydrogel dressing with macroporosity from printed geometry and the confocal image of emulsion-templated microporosity.

Typical bulk hydrogels are limited to geometries that can be cast into a mold. Recent research has focused on developing hydrogel inks with rheological properties suitable for 3D-printing to expand the geometries and architectures available.^49,50^ We have previously reported that hydrocolloid ink consisting of an aqueous solution of PEGDA emulsified with mineral oil exhibited high fidelity printing of complex shapes for rapid prototyping.^45^ A cure-on-dispense methodology was used to photo-crosslink the hydrocolloid ink during printing to generate an emulsion-templated hydrogel foam [Fig. 1(a)]. In the current study, we utilized this hydrocolloid ink to fabricate 3D-printed hydrogel dressings with the hierarchical porosity (Fig. 1). The lattice structure and geometry programed into the 3D-printing process provides the macroporosity, and the microporosity is generated by removing the oil droplets from dressing following polymerization of the continuous hydrogel phase [Fig. 1(b)].

**FIG. 2.**
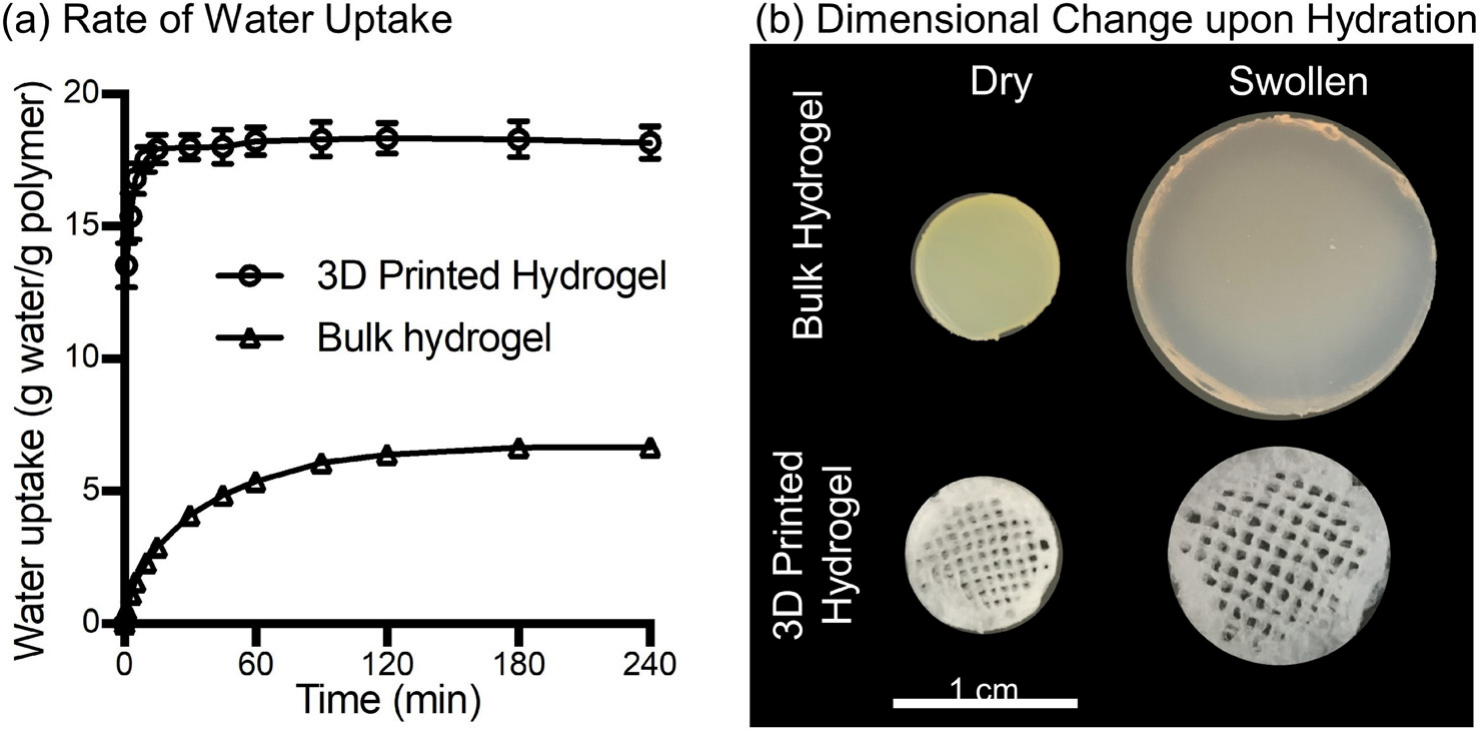
Characterization of 3D-printed hydrogel and bulk hydrogel. (a) Water uptake of the 3D printed hydrogel dressing and bulk hydrogel over 4 h. The time to reach 95% of equilibrium water uptake state is 15 min for 3D-printed hydrogel and 3 h for bulk hydrogel. (b) Representative images of dry and swollen specimens of 3D-printed hydrogel and bulk hydrogel.

**FIG. 3.**
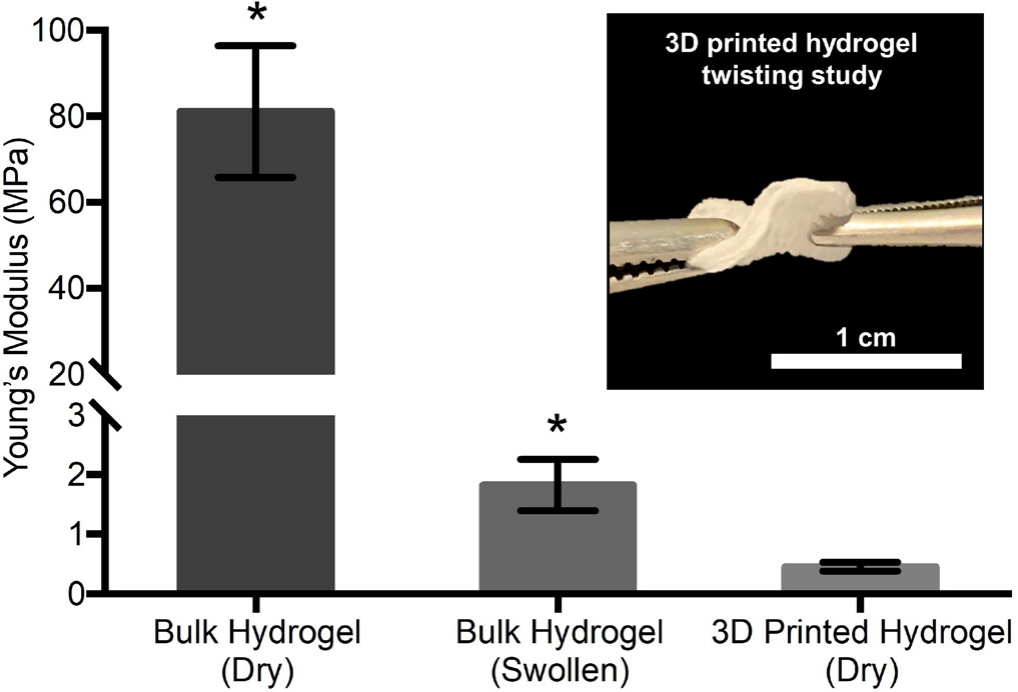
Comparison of flexural Young's moduli derived from standard 3-point bending tests according to American Society for Testing and Materials (ASTM) D790-03, of dry bulk hydrogel, hydrated bulk hydrogel, and dry 3D-printed hydrogel with inset images of test specimens. ^*^ Indicates a significant difference with each of other groups at p < 0.05 (unpaired Student's t-test).

After fabrication and oil removal from the 3D-printed hydrogel dressings, swelling kinetics, dimensional changes upon hydration, and mechanical properties were characterized in comparison to bulk hydrogel samples of similar chemical composition. The 3D-printed hydrogel dressings displayed rapid water uptake, reaching equilibrium swelling after 15 min compared to the bulk hydrogels that required 3 h to reach equilibrium swelling [Fig. 2(a)]. The increased rate of hydration was attributed to the hierarchical porosity that facilitated uptake of water by both capillary action and hydrogel absorption. Additionally, the printed hydrogel dressings were able to absorb over 3 times more water and exhibited less dimensional change upon hydration than the bulk hydrogels [Fig. 2(b)]. For mechanical characterization, three-point bending tests were performed to obtain the flexural Young's modulus. A statistically significant decrease in flexural Young's modulus was measured for 3D-printed hydrogel dressings compared to either dry or swollen bulk hydrogels (Fig. 3). In contrast to the rigid bulk hydrogels, the 3D-printed hydrogel dressings were flexible as demonstrated by a forceps-twisting test. No damage to the dressing was noted, and the dressing retained its shape and mechanical properties.

### B. *In vitro* bacterial inhibition and cellular response

**FIG. 4.**
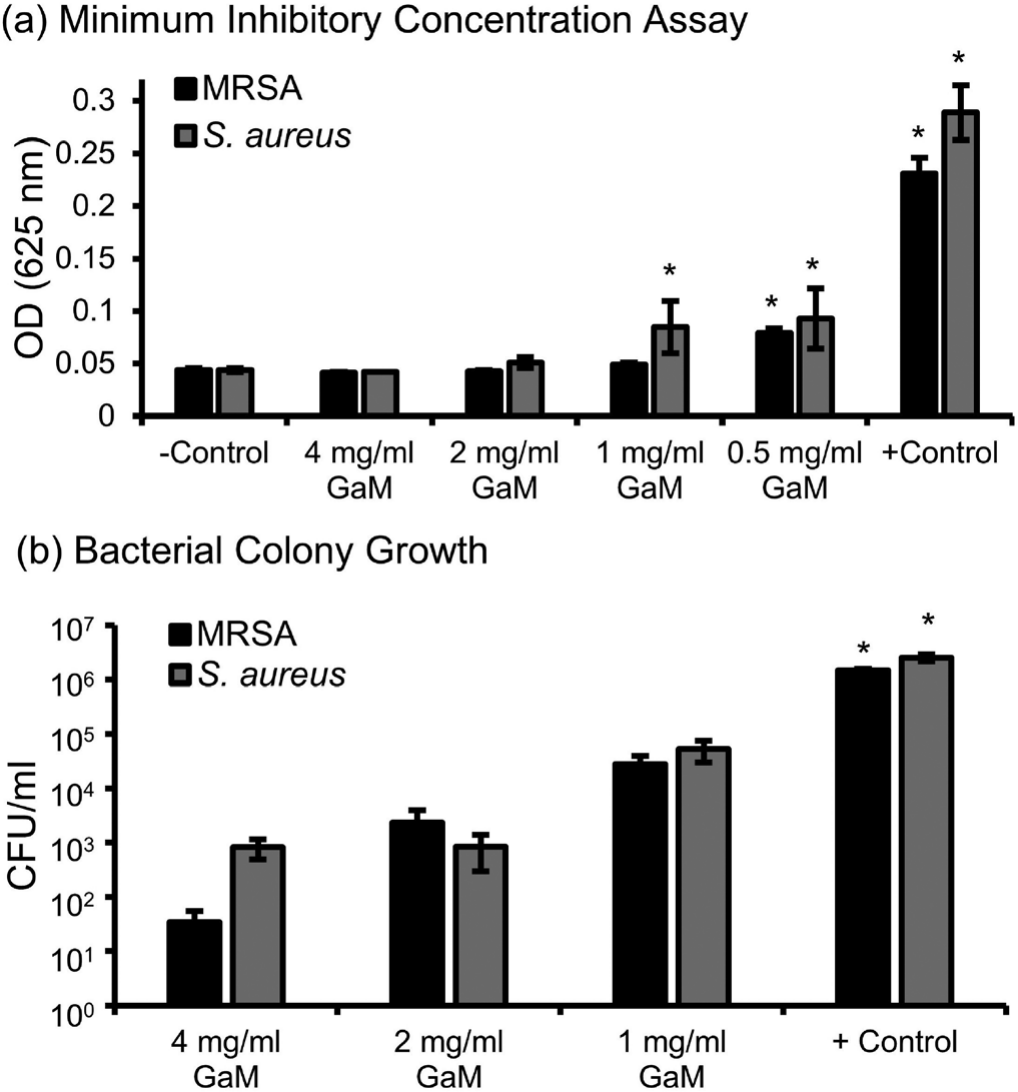
(a) Determination of minimum inhibitory concentration for GaM in MRSA and *S. aureus*. Minimum inhibitory concentration identified at 1 mg/ml and 2 mg/ml for MRSA and *S. aureus*, respectively. ^*^ Indicates statistical differences with respect to the negative control. (p < 0.05) (b) Bacterial colony growth after 24-h exposure to GaM at concentrations at and above at the MIC. ^*^ Indicates statistical differences with respect to the positive control (p < 0.05).

Bacterial inhibition studies were performed to identify therapeutic ranges of soluble GaM for both *Staphylococcus aureus* and methicillin-resistant *S. aureus* (MRSA). The minimal inhibitory concentrations (MIC) of *S. aureus* and MRSA were found to be 2 mg/ml and 1 mg/ml, respectively [Fig. 4(a)]. These reported concentrations had no visible growth of bacteria after 24 h compared to the negative control. These GaM MICs for *S. aureus* and MRSA were consistent with those reported previously by Baldoni *et al.*^39^ Optical density (OD) was utilized to determine statistical changes in bacterial growth and confirmed the visual MIC assay. To quantify bacterial growth after 24 h of exposure to GaM, colony forming units (CFU) for a single concentration below the MIC and up to 4 mg/ml were counted for both *S. aureus* and MRSA. A concentration dose dependence was demonstrated by reduction in CFU/ml as shown in Fig. 4(b). Concentrations below the MIC for both *S. aureus* and MRSA resulted in a significantly increased bacteria colony counts of ∼5.3 × 10^4^ and 9.4 × 10^4^ CFU/ml, respectively. Although these concentrations do not illustrate a bactericidal effect, MICs are the standard method for characterizing microbial susceptibility.^51^ Additionally, it has been shown that concentrations of bacteria less than 10^5^ CFU/g tissue allowed for wound healing to proceed normally.^52^ These results demonstrate the utility of GaM in reducing bacterial activity with potential to inhibit bacterial load *in vivo* at concentrations above the MIC.

**FIG. 5.**
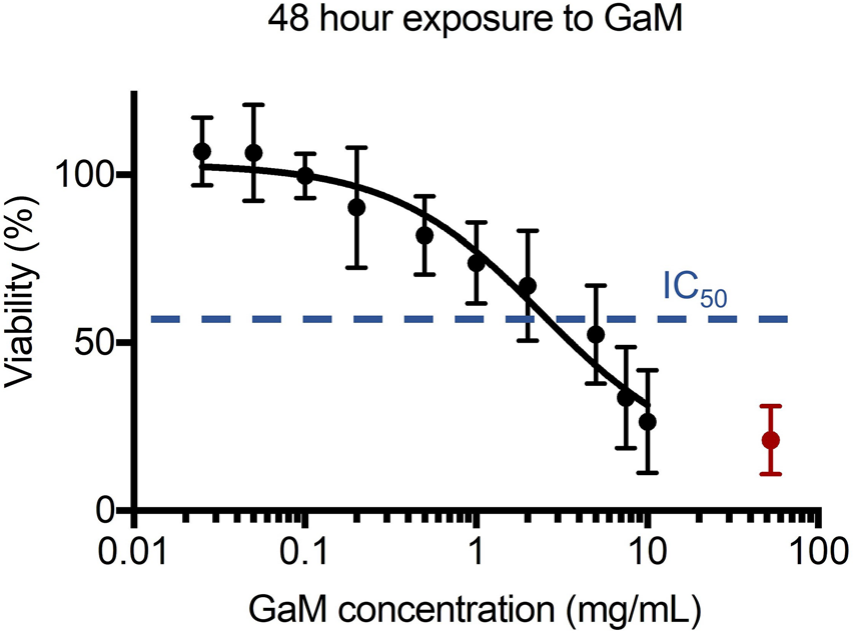
Relative viability of human dermal fibroblasts (hDFs) after 48-h exposure to different concentrations of GaM. A relative IC_50_ value was identified as 3.6 ± 1.2 mg/ml, noted by the blue dash line. The red symbol indicates the relative viability of the negative control group treated with 70% ethanol.

In addition to the bacterial inhibition, the effect of GaM on human dermal fibroblast (hDF) viability was investigated in a 48-h exposure study. The relative IC_50_ value of GaM for hDFs was identified as 3.6 ± 1.2 mg/ml (Fig. 5) with an associated selectivity index (IC_50_ divided by MIC) of 3.6 ± 1.2 (MIC = 1 mg/ml).

### C. Gallium maltolate loading and release

**FIG. 6.**
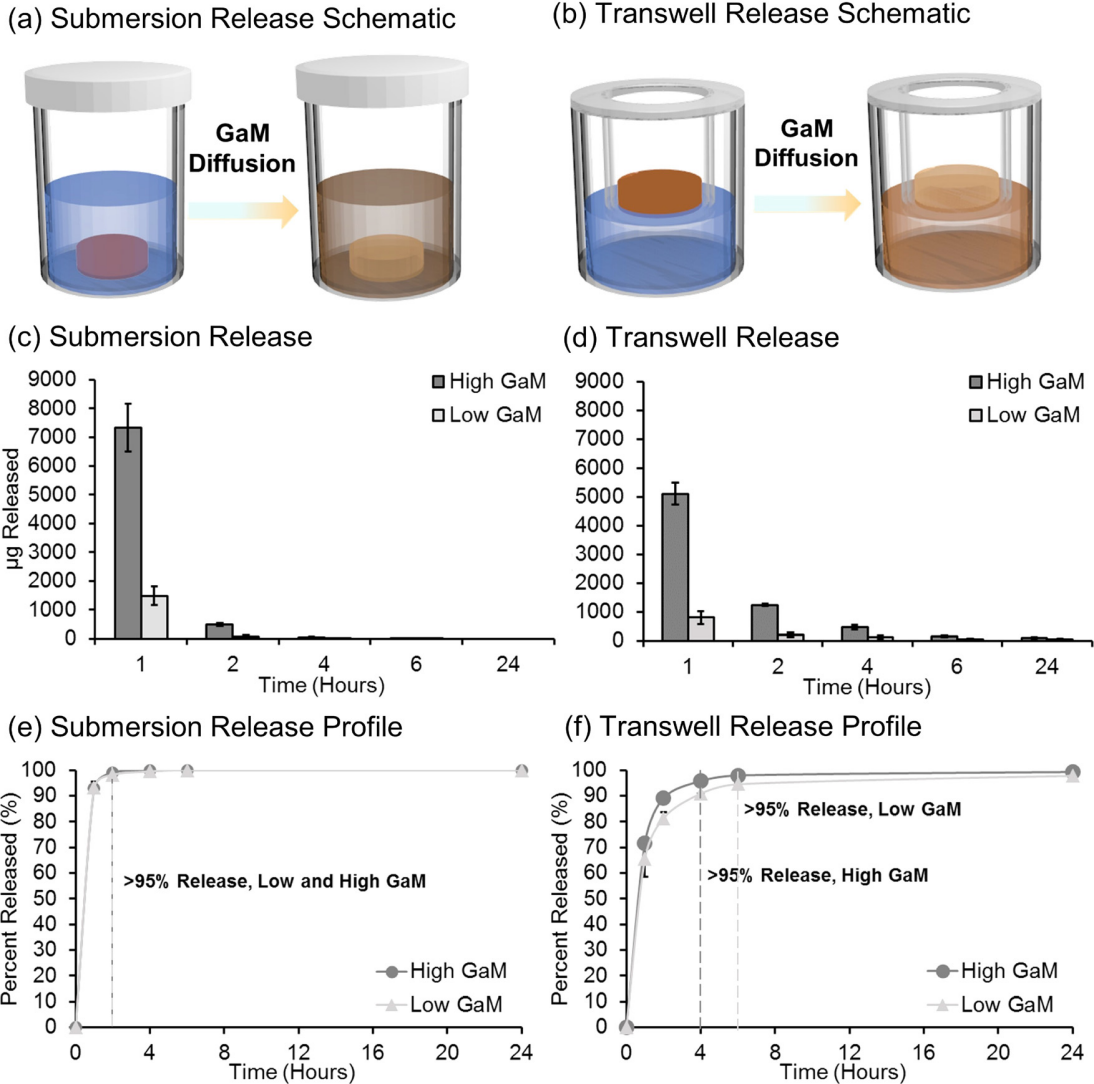
GaM hydrogel release schematic for 3D-printed hydrogel dressings in (a) Transwell and (b) the submersion model. *In vitro* GaM hydrogel release profiles from 3D-printed hydrogel dressing in (c) and (e) Transwell and (d) and (f) the submersion model.

As described previously, GaM was loaded into hydrogel dressings utilizing dichloromethane (DCM) to increase solubility of GaM and increase loading dose. GaM-loaded dressings were fabricated with a low GaM (1.4 ± 0.4 mg) and a high GaM (7.1 ± 0.7 mg) level, and the *in vitro* release profiles of GaM from the dressings were investigated (Fig. 6). The current method displayed low batch variability in GaM loading (supplementary material Fig. S3). If necessary, increased distribution and homogeneity can be improved using sonication or ultrasound in future iterations.^72^

Currently utilized release testing systems range in complexity ranging from diffusion cell models, organ-on-a-chip, and *in vitro* skin models.^53–56^ Ng *et al.* demonstrated variability in a static Franz diffusion cell system due to membrane barrier, sampling volume, and sampling frequency.^57^ Additionally, there has been extensive research investigating *ex vivo* animal and human models; however, several limitations exist due to concerns about ethics, hair density, and thickness.^54^ Here, we used two distinct release conditions, sink and diffusion methods, to characterize the release of GaM from our hydrogel dressings. First, a commonly utilized submersion method was investigated to create sink conditions to determine release rates [Fig. 6(a)]. As expected, submersion release profiles demonstrated a burst release of greater than 95% after 1 h, with concentrations below MICs after this time point [Figs. 6(c) and 6(e)]. Next, a diffusion release method was developed by placing 3D-printed hydrogel dressings into a Transwell^®^ insert and allowing diffusion of GaM molecules through a polyester insert membrane with 0.4-*μ*m pores [Fig. 6(b)]. Solutions were collected over 24 h to approximate antimicrobial release from the tissue contacting surface of the dressing. High burst release, greater than 65%, was observed after 1 h for both concentrations [Figs. 6(d) and 6(f)]. However, release concentrations were greater than the MIC for high GaM concentrations observed after 1 h for *S. aureus* and 2 h for MRSA. There was a minimal concentration-dependent effect observed with low GaM loaded dressings, as they retained similar release profiles over the 24 h. However, at this loading concentration, only the initial first hour time point resulted in concentrations meeting the MIC therapeutic range for MRSA.

**FIG. 7.**
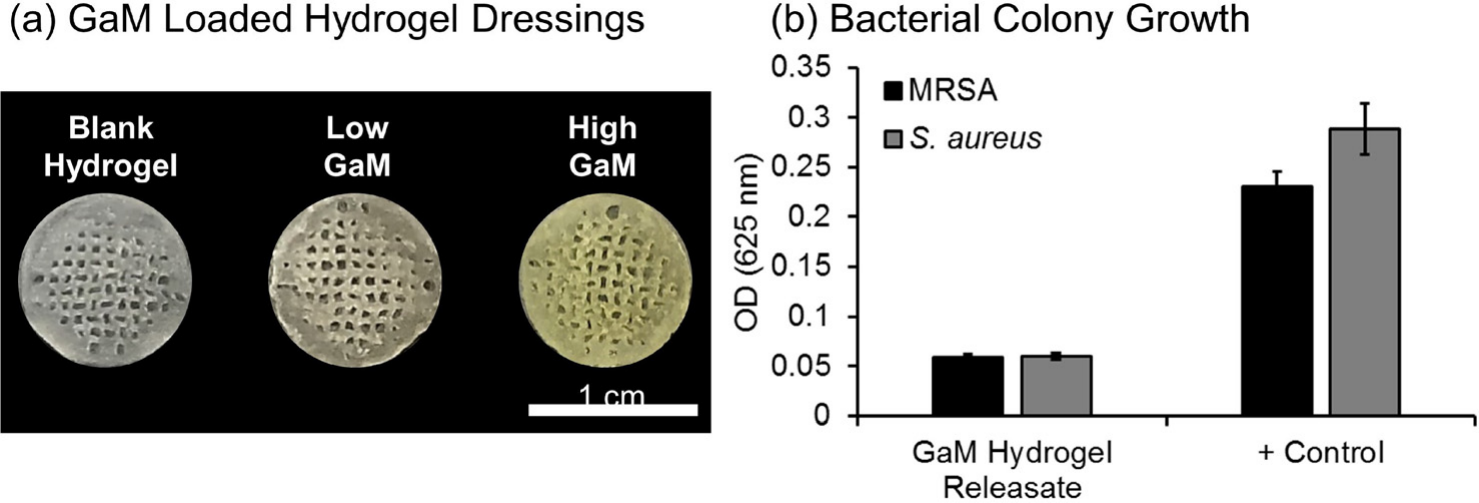
(a) GaM loaded hydrogel dressings with the increasing GaM amount. (b) Effect of GaM released from antimicrobial-loaded hydrogels on bacterial growth measured by changes in optical density.

To ensure that the GaM retained the antimicrobial activity after loading into 3D-printed hydrogel dressings [Fig. 7(a)], a modified MIC assay was performed to investigate bacterial growth in the presence of hydrogel releasates [Fig. 7(b)]. GaM was released from 3D-printed dressings by submersion in supplemented Roswell Park Memorial Institute (RPMI) media at a concentration of 4 mg/ml, which is above the MIC for both MRSA and *S. aureus*. Optical density analysis confirmed that bacterial growth was inhibited after 24 h with densities matching negative controls. This demonstrated that GaM retained its bactericidal properties after loading and release from the 3D-printed dressing.

### D. Murine splinted-wound model analysis

**FIG. 8.**
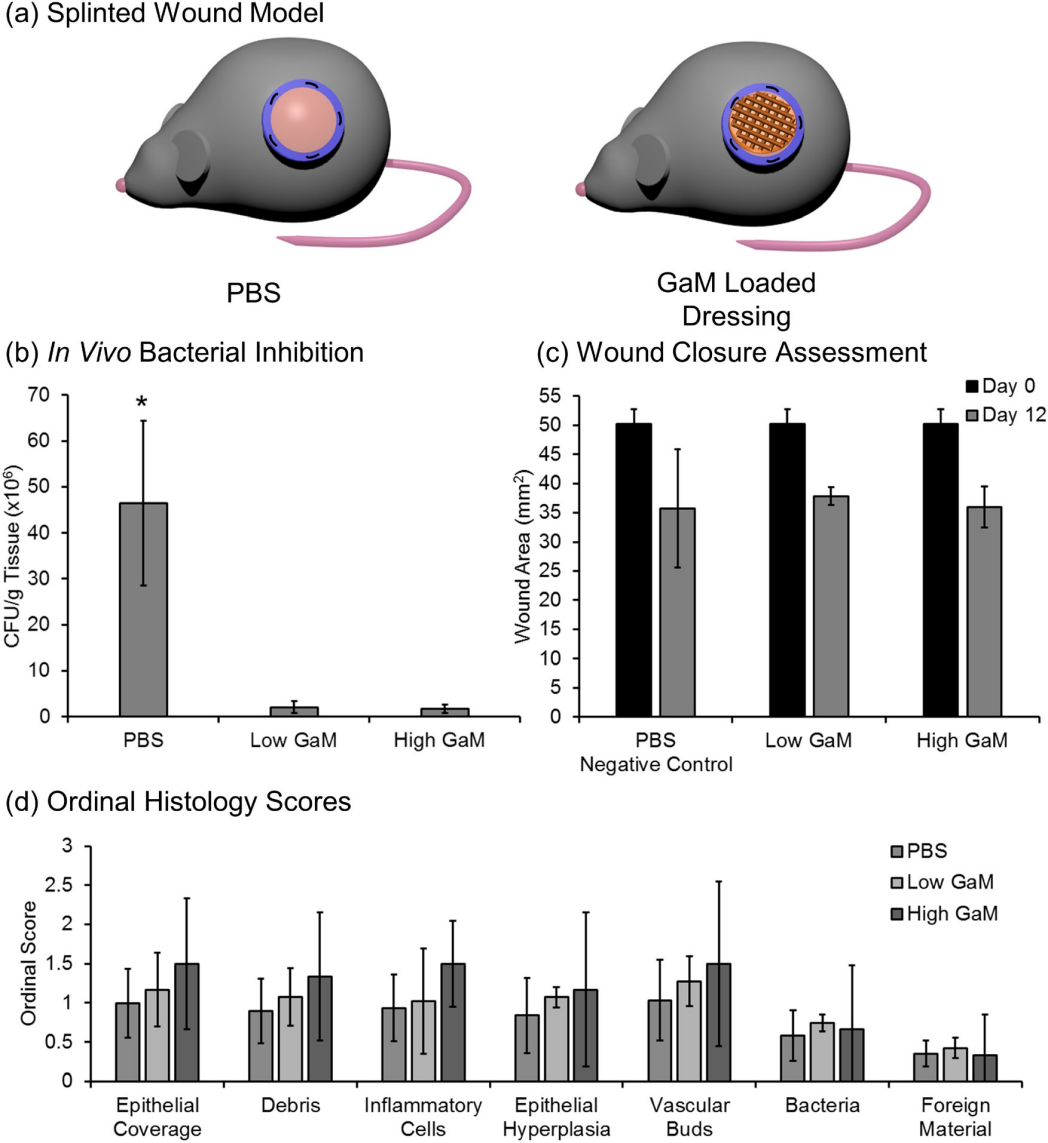
(a) Splinted murine wound model schematic with untreated control and applied 3D-printed dressing. (b) *In vivo* bacterial inhibition determined by CFU/g of tissue for low and high GaM loaded hydrogel dressings. (c) The wound closure assessment of all treatment groups at day 0 and day 12. (d) Ordinal histology scores investigating vascularization, inflammatory response, and wound closure. Data represented as average ± SEM. ^*^ Indicates statistical difference between corresponding samples (p < 0.05).

Splinted wounds were then investigated in a murine model to determine the effects of hydrogel dressing application on bacterial load and the rate of wound closure [Fig. 8(a)]. First, mass spectrometry was performed to quantify initial (0 h) and remaining (48 h) GaM concentration in the dressings after initial application. Initial GaM loading amount was determined to be 1597.7 ± 60 *μ*g and 5089.4 ± 952.3 *μ*g per dressing for the low and high GaM loaded dressings, respectively. Less than 3% of GaM remained after 48 h with 6.3 ± 4.1 *μ*g and 148.9 ± 116.2 *μ*g, remaining in the low and high dressings, respectively. These findings confirmed the *in vitro* burst release profile with full delivery of the loaded GaM within 48 h. Based on these findings, dressing changes were performed every 48 h to ensure GaM concentration was in the therapeutic range for 12 days. The use of these dressings as a carrier for GaM release resulted in a significant decrease in bacterial load in the infected wounds, ∼2 × 10^6^ CFU/g tissue compared to phosphate-buffered saline (PBS) treated controls, ∼50 × 10^6^ CFU/g tissue [Fig. 8(b)]. There was no significant difference in the wound closure rate of the untreated control and wounds treated with the GaM-loaded dressings with approximately 30% wound closure after 12 days [Fig. 8(c)]. Histological analysis determined no significant differences (p > 0.05) between the 2 GaM doses and the untreated control in terms of epithelial coverage [Fig. 8(d)]. This confirmation of wound closure via histological characterization further supports wound dimensional analysis. Additionally, there was no increase in the foreign material at the wound site as compared to the PBS control, indicating that there was minimal debris resulting from the dressing application and dressing changes. Boateng *et al.* reported that foreign bodies introduced into the wound can cause chronic inflammatory responses and lead to wound healing complications.^6^ Histological analysis of the wound site illustrates comparable wound healing in all treatment groups. Inflammatory responses were comparable with all treatment types as indicated by a mild to moderate inflammatory cell accumulation and similar levels of vascular budding. Collectively, these findings indicate that GaM delivery resulted in reduced bacterial growth with no negative effects on wound healing.

## III. DISCUSSION

It remains challenging to treat chronic wounds complicated with infection due to the complexity of the wound environment and balancing design goals of promoting wound healing and infection control. We aimed to develop an improved wound dressing that could enhance exudate management, provide wound moisture balance, and reduce the bacterial load. Maintaining a moist wound environment has been shown to facilitate the wound healing process by preventing tissue dehydration and cell death, enhancing angiogenesis, improving breakdown of necrotic tissue and fibrin, and initiating the interaction of chemotactic factors with target cells.^7^ However, excessive moisture can lead to delayed healing due to wound maceration and increases the frequency of dressing changes with the corollary increase in the cost of care. With low exudate, hydrogel dressings can maintain wound moisture and have a cooling effect. However, if the exudate exceeds the absorptive capacity of the hydrogel, maceration and delayed wound healing can result, and frequent dressing changes are required. Foam dressings are often used on moderately to heavily exudative wounds to manage exudate levels for less frequent and easier dressing changes but can lead to wound dehydration if the exudate becomes insufficient.^58,59^ Clinically used dressings such as Granugel^®^ and Aquaform^®^ have been shown to absorb 23%–27% in highly exudative wounds but only allow for a marginal 3%–5% hydration donation.^60^ We have developed a 3D-printed hydrogel dressing with the hierarchical porosity to combine the advantages of these two dressings with exudate management of foams and long-lasting hydration of hydrogels. The increased water uptake and rapid swelling of our 3D-printed dressings were attributed to the foam capillary action conferred by the hierarchical porosity. In contrast to other foam dressings, we hypothesize that the hydrogel matrix will provide sustained hydration for the improved moisture balance. Current studies are characterizing the water vapor transmission of these dressings over time and the benefit of this feature on cell behavior. Finally, this process is amenable to a broad range of hydrogel chemistries that can be used to meet application specific needs. For example, a biodegradable hydrogel matrix would eliminate concerns of residual foreign bodies within the wound bed.

In chronic wounds, issues with infection with biofilm development, excessive inflammatory responses, and the inability of cells to respond appropriately to reparative chemotactic factors prevent the phases of wound healing from occurring.^5^ Chronic wounds have complex colonizing bacteria with *S. aureus*, being the most prevalent in venous leg ulcers.^61^ In the late 1970s and early 1980s, the emergence of MRSA became an endemic nosocomial pathogen in the United States.^62^ *S. aureus* ATCC^®^ strain 43300 is resistant to all β-lactam antibiotics, demonstrating the complications with traditional antibiotic therapies.^63^ Antimicrobial resistance is a rapidly developing issue which could result in increased rates of morbidity and mortality.^64^ Gallium has the potential to overcome typical resistance mechanisms associated with antibiotics such as decreased cellular uptake due to permeability of the cellular envelope. Gallium mimics Fe(III) pathways in bacteria, which improves cellular uptake.^65,66^ The inability for Ga(III) to be reduced like Fe(III) interrupts the reduction and oxidation processes necessary for DNA and protein synthesis causing decreased cell proliferation.^67–69^ In addition to its established broad-spectrum bactericidal activity, topical application of GaM at low doses has been shown to reduce inflammation.^68,69^ Also, gallium has been shown to promote collagen synthesis and cell migration that could be beneficial for improved wound closure and healing.^43,44^ We hypothesized that a 3D-printed hydrogel dressing loaded with GaM would combine infection control and enhanced moisture balance and exudate management to improve *in vivo* wound healing. The local delivery of antimicrobials in this method is preferred over systemic delivery to treat infection to reduce toxicity, increase efficacy, and overcome problems associated with poor blood circulation in lower extremities commonly afflicting patients suffering from diabetes mellitus.^6^

The *in vivo* evaluation of GaM demonstrated improved infection control and comparable wound closure rates to that of the untreated control. The concentrations of GaM selected were shown to be within the therapeutic range for bacterial inhibition *in vitro* and displayed bactericidal activity in the murine splinted-wound model. In some studies, gallium-based compounds are utilized as anticancer treatments to induce apoptosis in cells; however, there were no negative effects on the inflammatory cell infiltrate or wound healing observed in the current study.^40,69–71^ In addition, the investigation of hDF viability following GaM exposure and calculation of the selectivity index (3.6 ± 1.2) indicated no adversary effect of GaM on cell viability at target bacterial inhibitory concentrations (2 mg/ml for *S. aureus* and 1 mg/ml for MRSA). The incorporation of GaM into this 3D-printed wound dressing provides an alternate method compared to topical delivery that suffers from limited control of dose and release kinetics, as well as poor tissue residence. Overall, these studies demonstrate the therapeutic potential of GaM delivery from a 3D-printed hydrogel dressing; however, there are several noted limitations that are the subject of future research. Most notably, the bacterial load was markedly reduced in the wounds treated with GaM; however, the bacterial load was not reduced below the critical threshold of less than 10^5^ CFU/g tissue that permits wound healing to proceed normally.^52^ GaM release was rapid from the hydrogel which necessitated frequent dressing changes. Although full release was confirmed at the 48-h dressing change, it is likely that the release was more rapid and the GaM concentration fell below the therapeutic value between dressing changes. *In vitro* release studies indicated that hydrogel dressings released over 90% of total loaded GaM in about 4 h. Current studies are investigating encapsulating GaM-loaded poly (lactic-co-glycolic acid) (PLGA) microspheres to provide sustained delivery of GaM within the therapeutic range to improve long-term bacterial inhibition.

## IV. CONCLUSIONS

The aim of this study was to develop an improved wound dressing platform through incorporation of the novel antimicrobial agent, GaM, in a 3D-printed hydrogel dressing. The hierarchical porosity of this 3D-printed hydrogel dressing enabled increased water uptake and more rapid moisture balance. The antimicrobial activity of GaM was characterized by identifying minimum bactericidal concentrations in *S. aureus* and MRSA. Release profiles of GaM-loaded 3D-printed hydrogel dressings were identified using submersion and Transwell release systems, and retention of antimicrobial activity post release was confirmed. Additionally, the effects of GaM loaded hydrogel dressings on wound healing and antimicrobial activity were investigated *in vivo* using a murine splinted-wound model. Mass spectrometry analysis was utilized to confirm complete delivery of therapeutic dosages prior to the dressing change. Explanted wound tissue confirmed decreased bacteria levels with the addition of the GaM loaded dressing and retention of wound closure rates. Overall, this work provides a versatile platform that can be used to provide a wound dressing matrix to support antimicrobial delivery and wound fluid balance in chronic wounds.

## V. METHODS

### A. Materials

All chemicals were purchased from Sigma Aldrich (Milwaukee, WI) and used as received unless otherwise noted. Trimethylolpropane ethoxylate triacrylate (TMPE, Mn = 912 Da), light mineral oil, and Kolliphor P188 surfactant were used in hydrocolloid ink formulations.

### B. Poly(ethylene glycol)-diacrylate synthesis

Poly(ethylene glycol)-diacrylate (PEGDA) was synthesized according to a method adapted from Hahn *et al.*^73^ Briefly, acryloyl chloride was added dropwise to a solution of PEG 2 kDa, 3.4 kDa, 6 kDa, or 10 kDa diol and triethylamine (TEA) in dichloromethane (DCM) under nitrogen. The molar ratio of PEG, acryloyl chloride, and triethylamine was 1:2:4, respectively. After the addition of acryloyl chloride, the reaction was stirred for an additional 24 h at room temperature. The resulting solution was then washed with 8 molar (M) equivalents of 2 M potassium bicarbonate to remove acidic byproducts. The product was then precipitated in cold diethyl ether, filtered, and dried under vacuum.

### C. Lithium phenyl-2,4,6 trimethylbenzoylphosphinate synthesis

Lithium phenyl-2,4,6 trimethylbenzoylphosphinate (LAP) was synthesized according to a method adapted from Fairbanks *et al.*^74^ Briefly, dimethyl phenylphosphonite was reacted with 2,4,6-trimethylbenzoyl chloride via a Michaelis-Arbuzov reaction. Equimolar amounts (0.006 mol) of 2,4,6-trimethylbenzoyl chloride was added dropwise to dimethyl phenylphosphonite with stirring at room temperature under a nitrogen blanket. The reaction mixture was stirred overnight, and then, a 4-fold excess (0.024 mol) of lithium bromide in 2-butanone (6 wt. %) was added to the reaction and heated to 50 °C for 15 min. The precipitated solid was then cooled to room temperature, filtered via vacuum filtration, and washed 3 times with 2-butanone.

### D. Bulk hydrogel fabrication

Hydrogel slabs (8 mm diameter, 1.5 mm thickness) were fabricated by making 25 wt. % precursor solutions of PEG(6K)DA and 5 wt. % TMPE crosslinker in de-ionized (DI) water. LAP photoinitiator (40% of total moles of acrylate groups) was added to the polymer precursor solution. Solutions were pipetted between 1.5-mm spaced plates and crosslinked by 6 min exposure to long wave UV light (Intelli Ray Shuttered UV Flood Light, Integrated Dispensing Solutions, Inc., 365 nm, 4 mW/cm^2^) on both sides.

### E. 3D-printed hydrogel dressing fabrication

Hydrocolloid inks were prepared using a FlackTek SpeedMixer DAC 150 FVZ-K as described previously.^45^ Prior to emulsification, PEGDA (25 wt. %) was added in DI water with Kolliphor P188 surfactant (10 wt. %), TMPE crosslinker (5 wt. %), and LAP photoinitiator (40% of total moles of acrylate groups) in the SpeedMixer cup. Once combined, light mineral oil was added to the aqueous, hydrogel solution in 4 additions and mixed at 2500 rpm for 2.5 min each, until a 75% weight fraction was achieved. Once emulsified, a ceramic stir-bead (10 mm diameter and height) was added and mixed at 3500 rpm for 2.5 min in the speed mixer.

3D-printed dressings were fabricated utilizing a RepRap Prusa i3 with an open-source RAMPS v1.4 electronics set and external MOSFETs to control the UV cure system. Hydrocolloids were loaded into a customized HYREL EMO-25 extruder equipped with a Luer lock adapter and a 22-gauge blunted stainless steel needle (413 *μ*m, 6.35 mm in length, Sigma Aldrich). The extruder was modified to print emulsion inks in a cure on dispense manner. Briefly, four 3-W ultraviolet (UV) LEDs (365 nm, Mouser Electronics, Mansfield, TX) were mounted to a heat sink and affixed to the extruder syringe, approximately 50 mm above the nozzle tip. The MOSFETs are externally powered to accept up to 24 V which allows for precise tuning of the voltage driving the UV LED cure source and allowed for UV of 100 mW/cm^2^. Cylindrical constructs (h = 4 mm, r = 10 mm) in OpenSCAD program were exported as an STL file and then imported into the “slicing” software, Slic3r version 1.2.9 with the following printing parameters: printing speed of 10 mm/s, nonprinting speed of 25 mm/s, layer thickness of 200 *μ*m, rectilinear grid infill of 70%, extrusion width of 0.6 mm, one perimeter, and no top or bottom solid layers.

The mineral oil was removed from printed constructs prior to characterization. The constructs were first allowed to completely air dry to allow for bulk oil removal and thorough swelling in DCM. Samples were then soaked in a series of washes for 1 h each in DCM, 50% v/v DCM/ethanol, ethanol, and 50% v/v ethanol/water. Finally, constructs were soaked overnight in water. After extraction and swelling in water overnight, constructs were frozen at −80 °C and lyophilized.

### F. Hydrogel characterization

Bulk hydrogel slabs and 3D-printed hydrogel dressings were characterized by measuring the water uptake over time and performing a three-point bending flexural test. For water uptake studies, hydrogel dressings were printed into cylinders with a diameter of 10 mm and a thickness of 2 mm. Following printing, the dressings were washed to remove mineral oil, swollen in water for 1 h to reach equilibrium swelling, frozen, and lyophilized. Bulk hydrogel slabs were fabricated (thickness = 1.5 mm), swollen in water for 3 h to reach equilibrium swelling, punched into 8-mm specimens, and then dried under vacuum overnight. The dried bulk hydrogels were trimmed to the same weights of the lyophilized hydrogel dressing specimens, and the dry weights of both groups were recorded (W_d_). Specimens were then submerged in DI water, and the swollen weights (W_s_) recorded at 1, 3, 5, 10, 15, 30, 45, 60, 90, 120, 180, and 240 min after submersion (n = 4). This experiment was repeated in triplicate (total n = 12). The water uptake (grams of water absorbed per g polymer) was calculated from the following equation:

$$\text{Water}\;\text{uptake}=\frac{W_{s}-W_{d}}{W_{d}}.$$ (1)

To compare the flexibility of bulk hydrogels and 3D-printed hydrogel dressings, three-point bending tests were performed using a dynamic mechanical analyzer (DMA-RSA3, TA instruments) according to the American Society for Testing and Materials (ASTM) standard D790–03.^75–77^ For testing, 25 mm × 10 mm rectangular specimens with 2 mm thickness of dry bulk hydrogel, swollen bulk hydrogel, and lyophilized hydrogel dressings were used to determine their flexural Young's modulus (E_flexural_) based on Eq. (2). The swollen hydrogel dressings were not evaluated due to low deformation forces that were outside the resolution of the load cell

$${E\;}_{\mathrm{flexural}}=\frac{L^{3}}{4h^{3}d}\times \frac{F}{\omega },$$ (2)

where L, h, and d are the effective length, thickness, and width of the specimen, respectively. F is the applied force and ω is the deflection. A deflection sweep with controlled displacements (ranging from 0.002 to 0.050 mm) was conducted, and the force required for the deflection recorded. This generates a force-deflection curve, and the slope (F/ω) of the linear region is inserted into Eq. (2) to obtain E_flexural_ (n = 3). Additionally, printed hydrogel dressings with the diameter of 10 mm and thickness of 2 mm were gripped with two forceps and twisted repeatedly to demonstrate their pliability.

### G. Gallium maltolate loading

Hydrogel dressings (D = 10 mm, T = 1.5 mm) were loaded at 2 concentrations of gallium maltolate (Gallixa LLC, Menlo Park, CA, USA): low (∼2 mg/dressing) and high (∼7 mg/dressing). To achieve these loading concentrations, GaM was dissolved in DCM at 8 mg/ml and 30 mg/ml to achieve low and high concentrations, respectively. Dried hydrogel dressings were measured (W_di_), placed into glass vials, and submerged in 2 ml of GaM solutions based on the desired concentration. Hydrogels were swelled for 3 h to reach equilibrium swelling to ensure full hydration in GaM solutions. Hydrogel dressings were then extracted and air dried for 10 min prior to an overnight vacuum dry. Dried hydrogel samples were then weighed (W_df_) to measure theoretical loaded GaM (M)

$$M=W_{\mathrm{df}}-W_{\mathrm{di}}.$$ (3)

Both low and high GaM loading were analyzed for 6 batches with one-way ANOVA to assess the batch variability, see supplementary material, Fig. S3.

### H. Gallium maltolate release

GaM release was performed under 2 conditions to better predict *in vivo* release, using submersion and Transwell models. In the submersion models, GaM loaded hydrogels were submersed in 2 ml of water, samples were extracted at distinct time points, and 2 ml of water was replaced. In the Transwell model, GaM loaded hydrogel samples were prehydrated with 80 *μ*l of RO water and placed into a 12 mm Transwell permeable membrane insert in a 12-well plate. Wells were filled with 500 *μ*l of water to fill up to the insert membrane interface. Releasate was collected at distinct time points, and water was replaced.

To measure GaM concentrations, a Cary 50 UV-Vis spectrophotometer (Agilent Technologies) recorded UV-Vis absorption spectra in the range of 200–400 nm. The data were collected with a scan speed of 300 nm/s and 0.5 nm resolution. Concentrations of GaM between 5 and 25 *μ*M were measured utilizing UV-Vis spectroscopy to create a calibration curve. A standard curve was developed utilizing linear regression analysis, and the unknown sample masses were then calculated, supplementary material Fig. S1. Collected GaM releasates were diluted to a theoretical concentration of 15–20 *μ*M with water to prevent saturation.

### I. Bacteria and growth conditions

*Staphylococcus aureus* (ATCC 29213™) and methicillin-resistant *S. aureus* (MRSA; ATCC 43300™) were cultured in brain heart infusion broth (BHIB; Beckton, Dickinson and Company, Sparks, MD, USA) for 24 h at 37 °C on a shaker plate at 250 rpm. Bacterial cells were pelleted by centrifugation at 3000 × g for 10 min and washed 3 times with phosphate-buffered saline (PBS; Thermo Fisher Scientific). The concentration of bacteria was determined spectrophotometrically (Smartspec 3000) at an optical density (OD) of 625 nm, and approximately 5 × 10^6^ colony forming units (CFU)/ml were inoculated into Roswell Park Memorial Institute 1640 Medium (RPMI; Thermo Fisher Scientific). All RPMI 1640 media were supplemented with 5 ml sodium pyruvate (100 mM, Thermo Fisher Scientific) and 5 ml GlutaMAX™ solution (200 mM, Thermo Fisher Scientific) per 500 ml RPMI 1640. RPMI was used as the control medium to assess the effects of GaM on growth of *S. aureus* and MRSA. In all experiments, concentrations of bacteria were determined by 10-fold serial dilutions cultured in triplicate on brain heart infusion agar.

### J. GaM minimum inhibitory concentration

The MIC of GaM against *S. aureus* and MRSA was determined by identifying the lowest GaM concentration that prevented visible bacterial growth. GaM was dissolved in RPMI at a concentration of 8 mg/ml. All MIC tests were performed using 96-well plates. All dilutions were 2-fold dilutions starting at 4 mg/ml GaM and ending at 0.25 mg/ml. 3D-printed hydrogels samples loaded with GaM were evaluated by submersion of dressings in RPMI to create a final concentration of 8 mg/ml, and releasate was evaluated at final concentration of 4 mg/ml. Wells containing RPMI medium with or without the *Staphylococcus* isolates were included as positive and negative control wells, respectively. Bacterial growth was measured by a change in turbidity of OD at 625 nm utilizing a microplate reader (BioTek Synergy 2) at time 0 and then at 24 h after incubation at 37 °C. After 24 h of GaM exposure, bacterial concentrations were determined and reported as CFU/ml.

### K. IC_50_ for human dermal fibroblasts and selectivity index of GaM

To evaluate the effect of GaM on human dermal fibroblasts (hDFs) and calculate the selectivity index (IC_50_/MIC), *in vitro* cytotoxicity assays (IC_50_) were performed with Promega's CellTiter 96^®^ AQueous One Solution Cell Proliferation Assay using a method reported in by Chua *et al.*^70^ Briefly, hDFs were seeded in 48-well plates at 10 000 cells/well and incubated at 37 °C for 24 h. GaM was dissolved in hDF culture medium at a series of concentrations from 0.025, 0.05, 0.1, 0.2, 0.5, 1, 2, 5, 7.5, to 10 mg/ml and added to the wells after sterilization by syringe filtering. Tissue culture polystyrene (TCPS) and 70% ethanol treatment were used as positive and negative controls, respectively. The colorimetric assessment of cell viability was measured at 490 nm after 48 h of GaM exposure. The assays were performed with 3 biological replicates with each replicate measured in triplicate. The IC_50_ value was generated with an embedded method “Dose response-inhibition” in Prism 7. Cell viability levels after exposure to various GaM concentrations were normalized to the positive control of TCPS viability and the negative control of ethanol treatment as the high and low boundaries of relative viability, rather than 100% and 0% viability. The selectivity index of GaM was calculated as the IC_50_/MIC.

### L. Murine splinted-wound model

Two-month-old C57BL/6 inbred mice were utilized to investigate bacterial inhibition and wound healing for a 3D-printed hydrogel dressing study. Mice were anesthetized with 3% isoflurane and injected with 0.3 mg/ml buprenorphine at a dose of 0.1 mg/kg. Their backs were shaved, and residual hair was removed with two applications of depilatory cream. The surgical area was then cleaned with chlorhexidine and isopropyl alcohol. One 8-mm biopsy punch was taken from the back, and a sterile silicone ring [10-mm outer diameter (OD) and 6-mm inner diameter (ID), 12-mm OD and 10-mm ID] was glued to the skin and sutured (Quill Monoderm VLM-1009) aseptically. Mice were inoculated with 30 *μ*l of 3.3 × 10^4^ CFU/ml of *S. aureus* (ATCC 29213) to yield a concentration of 1000 CFU/wound.

3D-printed hydrogel dressings (D = 10 mm, T = 1.5 mm) loaded with GaM were added to the mouse wounds 24 h after initial inoculation. GaM levels (Low—2 mg and High—7 mg) were chosen based on preliminary *in vivo* scouting studies and therapeutic range of *in vitro* MIC studies. 3D-printed hydrogel dressings loaded with GaM were sterilized via ethylene oxide sterilization. Treatment groups consisted of PBS, low, and high GaM-loaded dressings. GaM-loaded dressings were prehydrated with 80 *μ*l of PBS prior to application and rebandaging. Wounds were then bandaged with OpSite Flexifix bandages. Bandages and wound dressings were changed at 24 h after initial application and then every 2 days for 12 days. At each time, point bandages were removed, fresh dressings were applied, and animal body weights were obtained.

After 12 days, mice were euthanized, bandages and rings were carefully removed, and wound size was measured. Wounds were excised with 12-mm biopsy punches and split for analysis of bacterial growth and histology. Tissue excised for bacterial growth analysis was homogenized in 5 ml of PBS using a tissue homogenizer and diluted in PBS 1 × 10^2^-, 1 × 10^3^-, and 1 × 10^4^-fold. 100 *μ*l of diluted samples were plated in lysogeny broth (LB) agar plates and incubated at 37 °C for 24 h. Colonies were then counted to determine CFU/g of tissue. Tissues were processed for routine paraffin embedding, sectioned with a microtome (5-*μ*m-thick sections), and stained with hematoxylin and eosin (H&E). Samples were then scored based on criteria listed in supplementary material Fig. S2(d).

To quantify the *in vivo* GaM release, hydrogel dressings were collected at 48 h post application, and the GaM content remaining in the dressing was quantified using optical and mass spectrometry as compared to the initial concentration in dressings. Briefly, GaM-loaded specimens (0 h) were digested with nitric acid, hydrochloric acid, and hydrogen peroxide in a Milestone UltraWave microwave digester and then analyzed for GaM using Spectro CirOS and Perkin Elmer DRC 2 instruments. The initial GaM loading concentration of 4 specimens were reported as an average of quantified GaM mass. Hydrogel dressings (n = 6) were removed from the wound after 48 h and soaked in 70% ethanol. The liquid samples were then diluted with 1% nitric acid and the samples analyzed for GaM on a Perkin Elmer DRC 2 ICP-MS instrument. The GaM remaining in the dressing was reported as an average of GaM mass.

### M. Statistical analysis

All statistical analyses were expressed as the mean ± standard deviation unless stated as standard error of the mean (SEM). Statistical analysis was performed utilizing a standard 1-way ANOVA with Tukey's post-hoc analysis. Statistical significance was accepted at p < 0.05.

### N. Ethic approval

All procedures were approved by the Texas A&M University Institutional Animal Care and Use Committee (IACUC 2015-0383).

## SUPPLEMENTARY MATERIAL

See supplementary material for additional experimental data and information.
